# The Clinical Picture of Patients Suffering from Hypophosphatasia—A Rare Metabolic Disease of Many Faces

**DOI:** 10.3390/diagnostics12040865

**Published:** 2022-03-30

**Authors:** Izabela Michałus, Aneta Gawlik, Katarzyna Wieczorek-Szukała, Andrzej Lewiński

**Affiliations:** 1 Department of Endocrinology and Metabolic Diseases, Polish Mother’s Memorial Hospital-Research Institute, 93-338 Lodz, Poland; izabela.michalus@iczmp.edu.pl; 2 Department of Pediatrics and Pediatric Endocrinology, Faculty of Medical Sciences, Medical University of Silesia, 40-759 Katowice, Poland; agawlik@mp.pl; 3 Department of Endocrinology and Metabolic Diseases, Medical University of Lodz, 90-419 Lodz, Poland; katarzyna.wieczorek@umed.lodz.pl

**Keywords:** hypophosphatasia, rare disease, bone metabolic disease

## Abstract

Hypophosphatasia (HPP) is a rare, and usually diagnosed with delay, genetic disease caused by a mutation in the alkaline phosphatase liver/bone/kidney type (ALPL) gene. Low activity of the alkaline phosphatase (ALP) impairs the hydroxyapatite formation, reducing skeletal mineralization. The aim of the study was to present patients diagnosed with HPP. The data from the history and medical records of patients were analyzed. In the study group, one patient was diagnosed with perinatal type of HPP, three were diagnosed with infant variant, eight were diagnosed with children variant, two were diagnosed with odontohypophosphatasia, and two were diagnosed with the adult type of the disease. The most frequently presented symptoms included premature loss of teeth in 11/16 (68.75%) patients, bone deformities in 10/16 (62.5%) patients, chronic bone pain in 9/16 (56.25%) patients, and fractures in 8/16 (50%) patients. Reduction in bone mineral density in at least one examined projection has been found in 11/14 patients. Conclusions: The correct diagnosis of HPP is difficult due to the variety of types and clinical symptoms, as well as the very rare occurrence of this disease. Both lower and upper reference values of the determined biochemical parameters may be important in HPP diagnostics.

## 1. Introduction

Hypophosphatasia (HPP) is a rare genetic disorder caused by a mutation in the alkaline phosphatase liver/bone/kidney type (*ALPL*) gene [1,2,3]. The *ALPL* gene coding the tissue non-specific alkaline phosphatase (TNSALP) is located on the short arm of chromosome 1 (1p36.1–34) [1]. So far, more than 300 point mutations for this gene have been described [4,5,6]. The disease is inherited in an autosomal recessive or dominant manner, while the severity of symptoms depends on the mode of inheritance [1,2,3]. The incidence of HPP remains difficult to assess because of the lack of population studies.

Most publications cite the results from Canada, where the incidence of the disease is estimated at 1 in 100,000 live births (in Toronto, based on Ontario birth rate data), and data from France, where the prevalence of HPP is estimated as 1 per 300,000 for perinatal and infantile types [1,2,3,4,7]. The pathophysiology of the disease is related to the impaired function of osteoblasts that do not incorporate calcium into the matrix of the newly formed bone tissue.

The low activity of alkaline phosphatase (ALP) causes the accumulation of inorganic pyrophosphates in the extracellular fluid, which are inhibitors of skeletal mineralization and interfere with the formation of hydroxyapatite [1,2,3,4,7]. The clinical picture of the disease depends on the time of manifestation of symptoms. According to the severity of the symptoms, five types of HPP can be distinguished: perinatal, infantile, childhood, adult, odontohypophosphatasia, and pseudohypophosphatasia [1,3,7,8].

The most serious type is perinatal HPP, which is characterized by a deep skeletal hypomineralization that most often leads to intrauterine death of the fetus [1,2].

Prenatal and neonatal ultrasound images show deformations and shortening of the limbs, bone spurs, deformed chest, uneven calcification of the skull bones, and functional overgrowth of cranial sutures [9,10,11].

Frequently in the infants, vitamin B6-dependent convulsions and hematological disorders associated with the overgrowth of the marrow cavities in the bones are revealed [12,13]. Untreated children usually die within days or weeks of lung hypoplasia, which leads to respiratory failure [1,2]. The infant type appears in the first 6 months of a child’s life. The first symptoms are non-specific: reluctance for food and poor weight gain. Then, rickets-like symptoms are found, i.e., significant deformation of the chest and fractures of the ribs. One of the more characteristic symptoms during this period is premature overgrowth of cranial sutures—craniosynostosis. In biochemical studies, hypercalcemia and hypercalciuria are common, and they lead to nephrocalcinosis. The episodes of recurrent vomiting are also observed. The skeleton undergoes gradual demineralization, which is proven by the X-rays. The mortality from this disease in the first year of life is 50% [1,2,8]. The children’s type remains the most diverse in terms of symptoms. The first symptoms appear after 6 months of age. The premature loss of primary teeth (before the age of 5) is characteristic, and it usually takes place a few months after eruption.

Moreover, a delayed start of walking, unsteady gait, joint stiffness and pain, or muscle weakness (especially in the thighs) can be observed. All patients with this type of HPP have growth retardation, frequent fractures, and low bone mass. Premature overgrowth of cranial sutures may also lead to symptoms of increased intracranial pressure [1,2,8]. When HPP appears in adulthood, the premature loss of permanent teeth accompanies relatively good health. Stress fractures are frequent, especially in the area of the feet. The arthritis and degeneration of articular cartilage is caused by the deposition of inorganic pyrophosphates. However, an asymptomatic disease course, detected accidentally in laboratory tests, is also possible [1,14,15].

Odontohypophosphatasia manifests itself only within dental disorders, while pseudohypophosphatasia is characterized by clinical symptoms as in the infant type, with normal serum ALP activity [1].

The diagnosis of HPP is based on genetic, biochemical, as well as radiological studies of the skeleton. The serum contains a low level of alkaline phosphatase, while an increased concentration of inorganic pyrophosphates and phosphoethylamine is detected in urine [1,12,16,17]. In addition, biochemical tests may also show hypercalcemia, hyperphosphatemia, hypercalciuria, and a secondary reduction in parathormone (PTH) levels.

X-rays depict significant skeletal hypomineralization progressing over time, incomplete ossification of the vertebrae, bone spurs on the bones of the forearm, deformation of the chest, past fissures, and slowly healing fractures. Detecting the mutation in the *ALPL* gene confirms the disease [1,5,6,16].

Until recently, HPP remained a disease in which only symptomatic treatment was used: bone stabilization with rods, neurosurgical interventions in the case of craniosynostosis, orthodontic treatment and correction of biochemical disorders in the field of calcium–phosphate metabolism, as well as rehabilitation. However, the introduction of enzyme supplementation, dedicated to severe types that revealed in childhood, made it possible to improve the condition of patients, significantly alleviate their symptoms, and obtain better development and the quality of life [1,6,7,18,19].

## 2. Patients and Methods

### 2.1. Patients Included in the Study

We have assessed the data of 16 patients, aged from 1 month to 52 years (9 females and 7 males), including 12 children and 4 adults. The patients remained under the care of the Department of Endocrinology and Metabolic Diseases, the Polish Mother’s Memorial Hospital—Research Institute in Lodz, Treatment Centre for Osteoporosis and Other Metabolic Bone Diseases of in Children and Adolescents in Lodz, Foundation for Children with Musculoskeletal Disorders, Lodz, as well as Department of Pediatrics and Pediatric Endocrinology, Faculty of Medical Sciences, Medical University of Silesia in Katowice. All patients were diagnosed with HPP on the basis of clinical, biochemical, and radiological symptoms.

### 2.2. Clinical Examination

Clinical examination of the patients included primarily a medical history of symptoms from the skeletal system (bone pain, history of low-trauma fractures, skeletal deformities, abnormal gait, premature loss of deciduous teeth) and other symptoms characteristic for HPP, affecting the nervous system (vitamin B6-dependent seizures), respiratory system (respiratory failure), and the urinary system (nephrolithiasis). The patient’s age at diagnosis was also analyzed. On physical examination, psychomotor development, abnormal gait, and short stature were assessed. Attention was paid for skeletal signs characteristic of HPP (long bones and spine deformities, craniostenosis).

### 2.3. Biochemical Parameters

The results of biochemical tests of calcium and phosphate metabolism have been analyzed (the activity of ALP, serum calcium and phosphorus levels and excretion of these ions in a portion or daily urine collection, the concentration of the liver metabolite of 25-OHD and PTH). Serum concentrations of calcium, phosphorus levels, and ALP activity were determined using a routine analytical method. Serum values of 25-OHD and PTH were determined by using electrochemiluminescence detection (Cobas 6000—Roche, Basel, Switzerland).

### 2.4. Imaging Techniques

The X-ray of the skeleton was performed in case of bone deformities and/or suspected fractures—HPP-related radiographic abnormalities were analyzed. Abdominal ultrasound was routinely performed to evaluate the urinary system, with particular attention to features of nephrolithiasis.

In 14 patients, bone mineral density was assessed by the double-beam X-ray absorptiometry (DXA) using the same scanner (GE Lunar Prodigy) in total body mode (in children) or femoral neck (in adults) and spine (all ages). Z-score was calculated for children and T-score was calculated for adults.

### 2.5. Genetical Tests

In all patients, the diagnosis was confirmed by genetic testing (pathogenic mutation in the *ALPL* gene).

### 2.6. Ethics Committee Approval

The study was approved by the Bioethics Committee of the Polish Mother’s Memorial Hospital—Research Institute, Lodz, Poland (KB-80/2021).

## 3. Results

### 3.1. Clinical Phenotype

In the group of examined patients, the earliest diagnosis was made in a girl in the first month of life, while the latest was in a 52-year-old male. Most often, the disease was suspected earlier and then further diagnosed and confirmed by the results of genetic testing in a group of children between 2 and 6 years of age (nine patients). The perinatal type of HPP has been diagnosed in one patient, infantile type in three patients, childhood odontohypophosphatasia in two patients, and adult type in two patients. The most frequently observed symptoms included premature loss of deciduous or permanent teeth in 11/16 (68.75%) patients, bone deformities in 10/16 (62.5%), chronic bone pain in 9/16 (56.25%), fractures in 8/16 (50%), and gait disturbance in 6/16 (37.5%) patients.

Less frequent symptoms in the study group—characteristic of HPP—were: motor development delay in 5/16 (31.25%), nephrocalcinosis in 4/16 (25%), premature overgrowth of cranial structures in 3/16 (18.75%), and seizures in 2/16 (12.5%) patients.

The analysis of clinical symptoms in individual types of HPP showed that the more severe type of the disease, the more clinical symptoms occur and they appear in a greater percentage of patients. In the perinatal and infantile HPP types, the spectrum of symptoms is rich, and many of the above-mentioned symptoms occur in each child from the analyzed HPP types—all children had bone deformities, psychomotor retardation, and in the majority of them (75%, three out of four), there were such severe symptoms as fractures or nephrocalcinosis. For the perinatal type, seizures were also characteristic. In the childhood type, the symptoms were present in different configurations, but each child in this group presented at least one symptom, most children lost their deciduous teeth prematurely (six of eight patients), and the patients (five out of eight) reported bone pain; other common symptoms in this HPP type were fractures and bone deformities (in four of eight patients). Children with odontohypophosphatasia, apart from the loss of deciduous teeth, showed no other characteristic symptoms. In adults, the course of the disease was either asymptomatic or skeletal abnormalities were found.

In Table 1, we present the clinical symptoms relevant for further diagnosis.

### 3.2. Biochemical Phenotype

Among disorders of the calcium–phosphate metabolism, 15/16 patients had a reduced activity of ALP, in one patient, the activity remained at the lower limit of the normal range. Other biochemical abnormalities also included hypercalcemia in 10/16 patients, hyperphosphatemia in 10/16 patients, PTH decreased levels in 5/16 patients, deficiency of hepatic metabolite of vitamin D–25-hydroxyvitamin D (25-OHD) in 5/16 patients, hypercalciuria in 7/16 patients, and hyperphosphaturia in 6/16 patients.

Analysis of the laboratory results in relation to the different types of the disease showed that most calcium and phosphate disturbances occur in the perinatal and infantile types. Abnormalities in serum ionogram and urinary excretion of ions were equally present. In the childhood type, hypercalcemia and hyperphosphatemia were the most common. The vitamin D deficiency, present in the adult type, seems to be related to population deficiencies of this vitamin rather than to HPP.

The most important, often life-threatening biochemical disorders, occurred in patients with severe types of HPP: perinatal or infantile (Table 2).

### 3.3. Imaging and Genetic Testing

**Figure 1:**
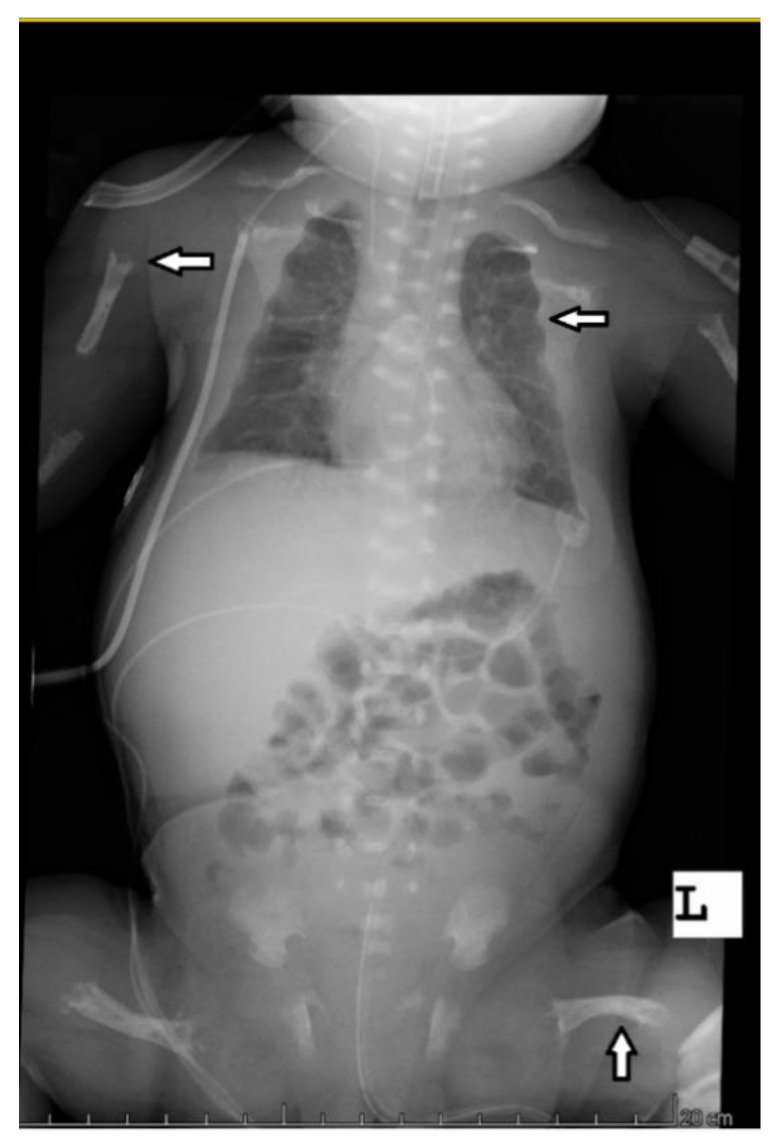
Babygram of a patient with the perinatal disease type—disturbances in bone mineralization, bone structure, uneven edges of the epiphyses, and bone deformities after fractures.

**Figure 2:**
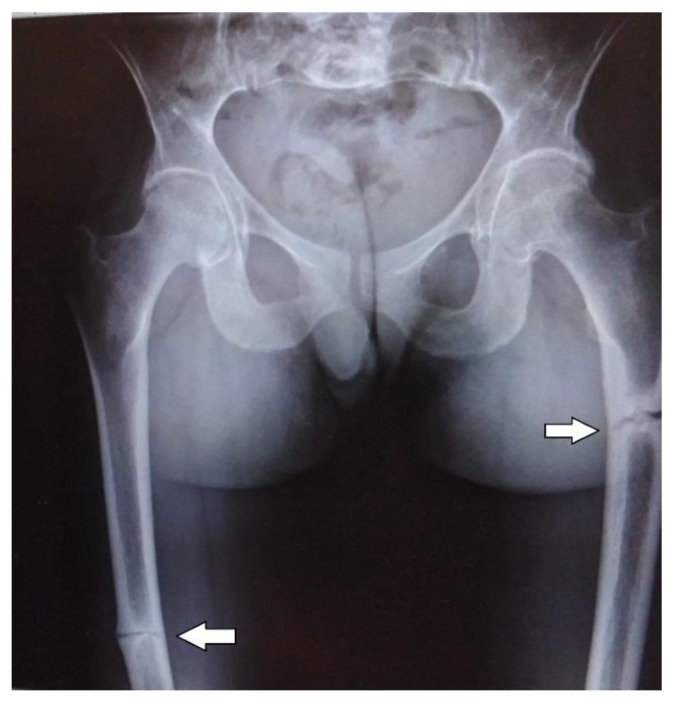
Fractures of the femurs in an adult patient with infantile HPP.

The abdominal ultrasound examinations showed the features of urolithiasis in 3/16 patients, and the X-ray examinations revealed the most common disorders of bone mineralization, abnormalities in the epiphysis of long bones–dilatation, uneven contours, bone defects, and fractures (Figure 1 and Figure 2).

DXA examination has been performed in 14 out of 16 assessed patients. In two children, the examination has not been performed due to age limitations. A decrease in bone mineral density in at least one examined projection has been found in 11 patients, where seven patients with the Z-score (in children) or T-score (in adults) values ranged from −1.1 to −2.0, and in four patients, values were less than −2.0 (Table 3). Genetic testing performed in all patients has confirmed mutations in the ALPL gene.

Bone structure abnormalities observed in X-ray were characteristic and most severe for the perinatal and infantile types (they occurred in all the patients of these groups), while in other HPP types, they occurred much less frequently. A decrease in bone mineral density, assessed by densitometry, was observed in the infantile and childhood types, but it should be noted that bone demineralization was described in the perinatal type on bone radiography. In this case, DXA was not performed due to the severe patient’s condition.

## 4. Discussion

Our study presents several cases of an extremely rare genetically determined disease, which is HPP. It is noteworthy that in the case of described patients, diagnosis—based on the clinical picture—took a lot of time, from 1 month up to 52 years, and the patients had been earlier consulted by the specialists in the field of endocrinology, nephrology, orthopedics, surgery, or dentistry. The test results and clinical symptoms suggesting HPP was found in five patients several months to several years before the diagnosis, but the clinical picture also suggested other possible entities involving skeletal deformities. In the HPP differential diagnosis, one should also take into account rickets, *osteogenesis imperfecta*, and other bone dysplasias, depending on the clinical type and dominant symptoms [3,20].

Accordingly, the patients presented by us had been diagnosed for *osteogenesis imperfecta*, achondroplasia, and other bone dysplasias. Similar observations were reported by Vogt et al. [21] in a retrospective evaluation of 50 patients with various types of HPP in children. They also noticed that in most cases, the diagnosis was made on the basis of clinical symptoms (88%) and/or decreased ALP activity (96%), which was also the case in our patients. What is more, the main clinical symptoms in the cited authors were the same as in our patients, i.e., premature loss of deciduous teeth, bone pain, and bone mineralization disorders; however, psychomotor development disorders were found more often [21]. It is to be recalled that in the study of Vogt et al. [21], the average age of patients at the time of diagnosis was 24 months, while we most often made the final diagnosis between the ages of 2 and 6, which means that the diagnosis in our department lasted longer.

In many reports describing HPP, the reason for the delay in diagnosis was lack of the interpretation of the result of the ALP activity test, which was always reduced, even if it accompanied poor clinical picture. It resulted not only from the opinion that exclusively high values of ALP represent an expression of disorders in the skeletal system but also from the lack of lower reference values for ALP. Similarly, in all our patients, the activity of ALP was borderline or below the normal range values. Interestingly, the most symptoms and biochemical disorders were presented by patients with perinatal and infantile disorders, and the most sparse symptoms were seen in adults and odontohypophosphatasia.

The authors of studies on adult HPP indicate non-specific symptoms of the disease: chronic fatigue, joint pain, microfractures, pathological fractures, especially of the femur, metatarsal bone fractures, but there are also patients with an asymptomatic course of HPP [22,23,24]. In two (2) adult patients presented in our study, the diagnosis was made after a genetic test in the child confirming a mutation in the *ALPL* gene. In one (1) patient, the analysis of the history showed the presence of skeletal symptoms—mainly bone pain and deformities, as well as enamel disorders of permanent teeth, while in the other adult patient, the disease was asymptomatic, and ALP activity was below the reference value in both of them. The delay in diagnosis was noted by Hogler et al. [25], who analyzed data from a prospective study of 269 patients from 11 countries included in the HPP registry. They found that in the group of children, clinical symptoms preceded diagnosis by more than 12 months, and in the adult group, the difference between the onset of symptoms and diagnosis was much longer—around 10 years. In the same study, the most common symptoms suggesting HPP were loss of deciduous teeth, bone deformities, and developmental delay. Most of the patients in the diagnosis of skeletal disorders had ALP measurements performed. However, in many cases, the lack of the lower range of the normal values or the normal values ranges inconsistent with the patient’s age resulted in a delay in the diagnosis.

Among the patients presented in our study, in at least four children, the examination did not lead to diagnosis (no lower reference values or no doctor’s response to reduced ALP activity values). In one adult patient, the diagnosis was not made due to the borderline values of alkaline phosphatase, despite the clinical symptoms lasting for over 50 years.

Diagnostic difficulties were also described in the study by Mohn et al. [26], where the diagnosis of HPP was made only in an 11-month-old infant, as the initial symptoms suggested deficiency rickets (large dimensions of the anterior parietal area). The patient was treated with increasing doses of 25-OHD and calcium supplements, which resulted in biochemical disorders at 8 months of age, and the supplementation was ceased due to hypervitaminosis D, hypercalcemia, and hyperphosphatemia. It was not until 11 months of age that further symptoms appeared—features of increased intracranial pressure, loss of deciduous teeth, and nephrocalcinosis, on the basis of which HPP was suspected. A completely different course of the infant disease type was described by Demirbilek et al., and in the patient described by these authors, the initial symptoms suggested neurological disorders [12]. From the first days of life, the child showed reduced muscle tone and convulsions; therefore, anticonvulsant treatment was started. No skeletal abnormalities were observed.

In the 4th month of life, the symptoms appeared, such as raised, pulsating crown, and shortening and bending of the bones of the limbs. In addition, numerous biochemical symptoms were noted, which allowed the initiation of diagnostics toward HPP. At that time, a very low activity of ALP, hypercalcemia, and decreased PTH concentration was found.

Within the group of patients described in our study, only two (2) children developed seizures. One (1) had seizures in the course of severe perinatal disease diagnosed in the neonatal period, and in the other, it was the only clinical symptom of HPP; therefore, the diagnosis was made only in the 5th year of life. It is noteworthy that the child had previously undergone ALP activity tests, but due to the lack of a lower reference range, there was no possibility to establish the diagnosis. In recent years, the results of treating patients with enzyme replacement therapy have also been published, describing the improvement in the condition and activity of patients [6,18,19]. Among the patients described by us above, three patients has been also treated this way.

Analysis of data of patients with HPP according to clinical types showed the well-known fact that the clinical symptoms, calcium–phosphate abnormalities, and abnormalities in X-ray images, are most frequently demonstrated by patients with perinatal or infantile type of HPP.

As can be seen from the data presented in this paper, patients with HPP require multidisciplinary care. Daniel et al. [27] discussed this problem in their publication, presenting patients with different types of HPP. Specialist care for patients varied depending on the nature of the symptoms and their severity; however, each patient usually required the care of several specialists [27].

Summarizing, we attempted at presenting the clinical picture of HPP in a group of patients with symptoms of calcium and phosphorus disorders as well as skeletal deformities. Our intention was to draw attention to the need for a thorough interpretation of biochemical tests, in particular the activity of ALP—in the case of which not only elevated but also decreased values may be a symptom of the disease. It is also essential to stress that the clinical course of HPP is very diverse, resembling other diseases of the skeletal system, which frequently results in a delayed diagnosis or even treatment errors.

## 5. Conclusions

1. The diagnosis of HPP is difficult and often delayed due to the variety of clinical types and symptoms as well as rare occurrence of this disease.
2. Diagnosing disorders that involve changes in the skeletal system, both lower and upper reference values of the determined biochemical parameters, should be taken into account. Their thorough analysis is the basis for the differential diagnosis.
3. Determination of ALP activity should be a routine test in patients with complaints and symptoms of the osteoarticular system but also in patients with neurological symptoms (especially convulsions) and nephrocalcinosis.
4. Patients with HPP require multidisciplinary care and the cooperation of specialists in diagnostics and treatment.

## Figures and Tables

**Table 1:** Age of diagnosis, type of HPP, and clinical symptoms in the examined patients.

Patient No.	Age of Diagnosis	Type	Clinical Symptoms								
			Fractures	Bone Deformities	Developmental Delay	Teeth Loss	Craniosynostosis	Bone Pains	Gait Disturbance	Nephrocalcinosis	Seizures
1	1 month	Perinatal	yes	yes	yes	NA	yes	NA	NA	no	yes
2	5 months	Infantile	no	yes	yes	yes	no	no	NA	yes	no
3	2.5 years	Infantile	yes	yes	yes	yes	yes	yes	yes	yes	no
4	15 months	Infantile	yes	yes	yes	yes	yes	yes	yes	yes	no
5	6 years	Childhood	no	no	no	yes (12 months)	no	no	no	no	no
6	3 years	Childhood	no	no	no	yes (13 months)	no	yes	no	no	no
7	2 years	Childhood	yes	yes	yes	yes	no	yes	yes	no	no
8	5 years	Childhood	no	yes	no	yes (18 months)	no	yes	yes	no	no
9	11 months	Childhood	yes	yes	no	yes (3 years)	no	yes	no	no	no
10	5 years	Childhood	no	no	no	no	no	no	no	no	yes
11	52 years	Childhood	yes	yes	no	no	no	yes	yes	yes	no
12	3 years	Childhood	yes	no	no	yes (2.5 years)	no	no	no	no	no
13	2 years 5 months	Odonto	no	no	no	yes (17 months)	no	yes	no	no	no
14	2 years	Odonto	no	yes	no	yes (1.5 years)	no	no	no	no	no
15	41 years	Adult	yes	yes	no	no	no	yes	yes	no	no
16	40 years	Adult	no	no	no	no	no	no	no	no	no

NA—not applicable.

**Table 2:** Results of laboratory (biochemical and hormonal) tests in all the patients included in the study.

Patient No.	Laboratory Tests							
	ALP (U/l)	ALP Reference Values (U/l)	Calcium Serum (mmol/L)	Phosphorus Serum (mmol/L)	PTH (pg/mL)	25-OHD (ng/mL)	Ca/Kr Urine	P/Kr Urine
1	5 ↓	48–406	3.31 ↑	3.39 ↑	4.6 ↓	19.19 ↓	3.0 ↑	2.5 ↑
2	26 ↓	82–383	3.96 ↑	4.75 ↑	3.0 ↓	41.5 N	2.22 ↑	1.43 ↑
3	50 ↓	108–317	2.6 ↑	2.26 ↑	6.56 ↓	28.9 ↓	0.87 ↑	1.06 N
4	24 ↓	38–126	2.4 N	1.68 N	35.7 N	36.2 N	1.2 ↑	0.6 N
5	65 ↓	156–386	2.51 N	1.57 N	27.0 N	23.1 ↓	0.07 N	1.50 ↑
6	62 ↓	134–346	2.65 ↑	1.98 ↑	11.6 ↓	41.98 N	0.02	1.97 ↑
7	20 ↓	120–488	2.46 N	1.57 N	23.0 N	36.7 N	0.17 N	1.01 N
8	22 ↓	116–483	2.49 N	2.11 ↑	26.3 N	36.6 N	No data	No data
9	26 ↓	116–515	2.56 ↑	1.89 N	23.7 N	35.2 N	0.49 ↑	No data
10	87 ↓	134–346	2.56 ↑	2.06 ↑	34.9 N	33.7 N	No data	No data
11	38 N	30–120	2.39 N	0.72 ↓	40.97 N	31.4 N	N	N
12	85 ↓	108–317	2.59 ↑	1.81 N	No data	No data	0.26 ↑	No data
13	40 ↓	108–317	2.61 ↑	2.07 ↑	4.89 ↓	52.8 N	0.16 N	1.12 ↑
14	21 ↓	134–346	2.51 N	2.04 ↑	22.1 N	No data	0.22 N	2.61 ↑
15	28 ↓	38–126	2.56 ↑	1.46 ↑	55.1 N	29.6 ↓	0.27 ↑	0.81 N
16	20 ↓	38–126	2.47 ↑	1.26 ↑	No data	22.9 ↓	No data	No data

**Table 3:** Results of imaging, densitometric tests, and genetic testing in all the examined patients.

Patient No.	Imaging Tests								Genetic Testing —*ALPL* Mutation
	Skeleton X-ray	Abdominal Ultrasound	DXA TB BMD	DXA TB Z-Score	DXA Spine BMD	DXA Spine Z-Score	DXA Neck BMD	DXA Neck Z-Score	
1	Epiphyses with unequal outlines, fractures of both forearms		Not performed						yes
2	Features of heterogeneous, generalized, thinning, and atrophy of the bone structure of the distal metaphyses and ossification nuclei of both femurs and proximal metaphyses of the tibia and fibula.	Nephrocalcinosis	Not performed						yes
3	Bones less calcified, epiphyses widened with unequal outlines with numerous bone lesions	Nephrocalcinosis	0.400	−2.3	0.465	−2.5			yes
4	Bone lesions and widening of epiphyses of long bones, features of past fractures after birth				1.078	−0.6	0.519	−3.8	yes
5			0.546	−1.5	0.678	−0.1			yes
6			0.419	−0.97					yes
7			0.588	−0.7	0.606	−1.0			yes
8			0.681	−1.9	0.729	−1.8			yes
9			0.544	−2.8	0.543	−2.7			yes
10			0.48	−1.7	0.479	−0.5			yes
11	Scoliosis, demineralization of vertebrae	Nephrocalcinosis			0.133	−9.2	0.473	−2.6	yes
12					0.445	−1.1			
13	Thigh and tibia massive, layers of the last growth with uneven outlines, wide with a rebuilt structure with foci of thickening and thinning		0.622	1.23					yes
14			0.393	−1.34					yes
15				−0.9				−1.6	yes
16			1.117	0.2			0.761	−1.2	yes

## Data Availability

The datasets used and/or analyzed within the framework of this study are available from the corresponding author on reasonable request.
